# Leptin receptor antagonist attenuates experimental autoimmune thyroiditis in mice by regulating Treg/Th17 cell differentiation

**DOI:** 10.3389/fendo.2022.1042511

**Published:** 2022-10-20

**Authors:** Wei Wang, Bo-Tao Zhang, Qi-Lan Jiang, Han-Qing Zhao, Qin Xu, Yang Zeng, Jia-Ying Xu, Jun Jiang

**Affiliations:** ^1^ Department of General Surgery (Thyroid Surgery), the Affiliated Hospital of Southwest Medical University, Luzhou, China; ^2^ Department of Clinical Nutrition, the Affiliated Hospital of Southwest Medical University, Luzhou, China; ^3^ Department of Orthodontics, the Affiliated Stomatological Hospital of Southwest Medical University, Luzhou, China

**Keywords:** leptin, experimental autoimmune thyroiditis (EAT), JAK2/STAT3 pathway, leptin receptor antagonist, NOD/ShiLtJ mouse

## Abstract

Leptin has been found to be involved in the development and progression of many autoimmune diseases. As an organ-specific autoimmune disease, the pathogenesis of Hashimoto’s thyroiditis has not been fully elucidated. It has been reported that serum leptin level is increased in Hashimoto’s thyroiditis, but other studies have not shown any difference. We replicated a mouse model of experimental autoimmune thyroiditis (EAT) with a high-iodine diet and found that injection of the leptin receptor antagonist Allo-aca reduced thyroid follicle destruction and inflammatory cell infiltration in EAT mice, and thyroxine and thyroid autoimmune antibody levels. Further investigation revealed that Allo-aca promotes the differentiation of Treg cells and inhibits the differentiation of Th17 cells. We believe that Allo-aca can alter the differentiation of Treg/Th17 cells by inhibiting the leptin signaling pathway, thereby alleviating thyroid injury in EAT mice. Interfering with the leptin signaling pathway may be a novel new approach to treat treating and ameliorating Hashimoto’s thyroiditis.

## Introduction

Leptin is a peptide hormone produced by adipose tissue that plays a key role in energy metabolism, growth and development, and regulation of the endocrine system (1–3). In the last 20 years, leptin has been found to play an important role in the regulation of autoimmune responses. It enhances the proliferation and differentiation of various immune cells such as T cells, B cells, and dendritic cells, and stimulates the production of pro-inflammatory cytokines (4–7). It has been reported that leptin is associated with the pathogenesis of systemic lupus erythematosus, multiple sclerosis (MS), and experimental autoimmune encephalomyelitis (EAE). It influences the occurrence and development of the diseases by altering the differentiation and proliferation of CD4+ T cells (8–11).

Hashimoto’s thyroiditis (HT), also known as chronic lymphocytic thyroiditis, is an organ-specific autoimmune disease caused by genetic, autoimmune, and acquired environmental factors. It is characterized by goiter, lymphocytic infiltration, and elevated serum thyroid autoantibodies, which are often accompanied by changes in thyroid function in the later stages of the disease. Previous reports have shown that autoreactive CD4+ T cells against thyroid antigens are particularly important in the development of thyroiditis, especially the imbalance of immune responses between Th1 and Th2 helper cells involved in the initiation and development of HT (12, 13). Some recent studies have described that the increased Th17 cells and the decreased Treg cells in CD4+ T cells may be involved in the pathogenesis of HT (14–16). Until now, little is known about the role of leptin in the pathogenesis of Hashimoto’s thyroiditis.

In this study, we hypothesized that leptin might participate in the occurrence and development of HT by regulating the differentiation of Treg/Th17 cells. We replicated the animal model of autoimmune thyroiditis (EAT) with female non-obese diabetes (NOD) mice (17, 18). The leptin signaling pathway was inhibited by injection of the leptin receptor antagonist Allo-aca to investigate the relationship between leptin and the pathogenesis of Hashimoto’s thyroiditis.

## Material and methods

### Experimental autoimmune thyroiditis mouse model

**Figure 1:**
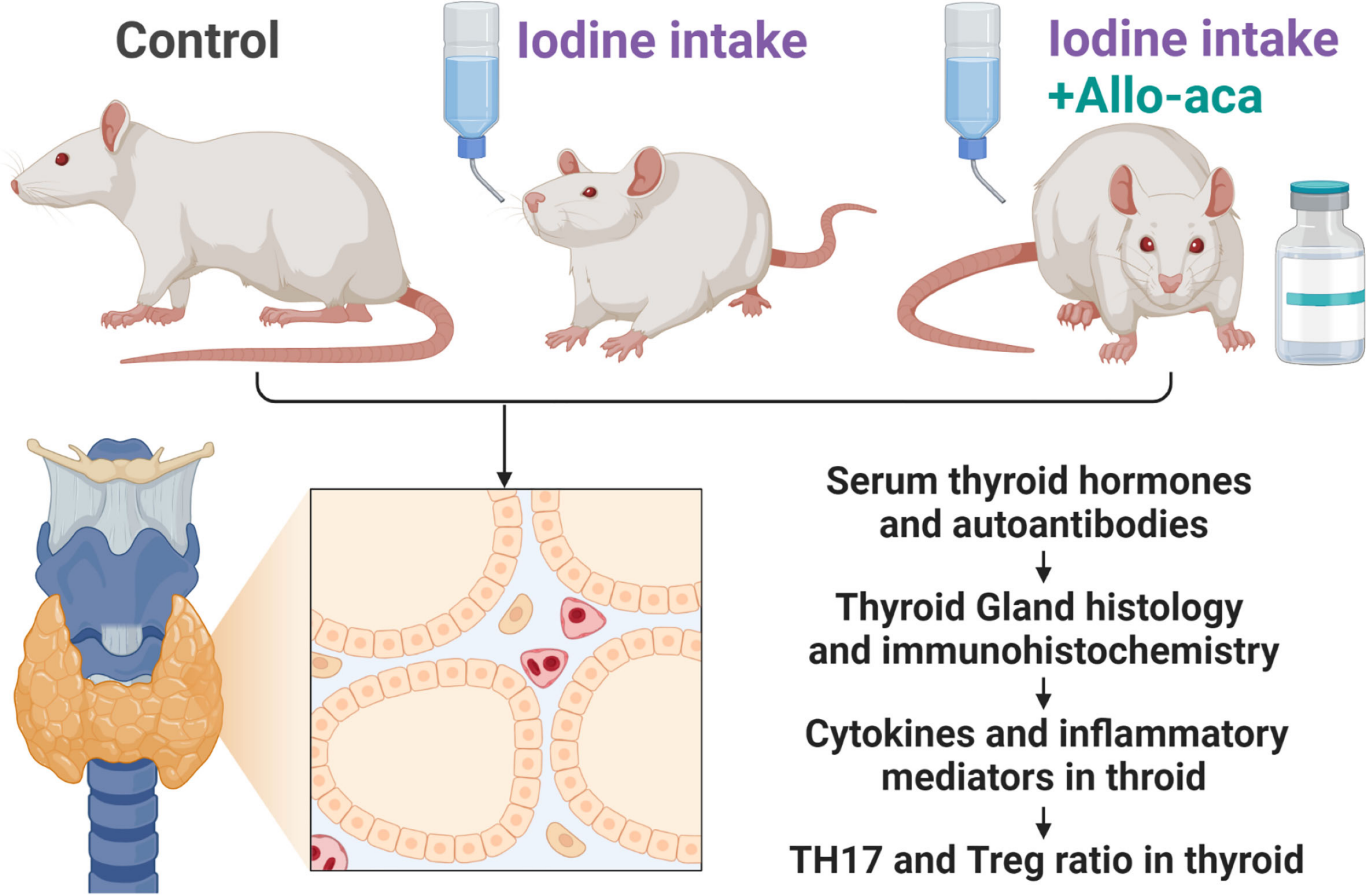
Experimental Procedure. NOD mice were divided into the control group, the experimental autoimmune thyroiditis (EAT) group and the Allo-aca treatment group. The EAT model was replicated by 0.05% iodine water intaking for 8 weeks. The Allo-aca treatment group received a subcutaneous injection of Allo-aca daily. The peripheral blood, thyroid and spleen of the experimental animals were used in the subsequent experiments.

The experimental process complied with the China Animal Management Regulations (Document No. 55 of the Ministry of Health of China in 2001) and was approved by the Animal Protection and Utilization Committee of Southwest Medical University (No. 20210827-008). Four-week-old female NOD/ShiLtJ mice (GemPharmatech, Jiangsu, China) were raised in a specific pathogen-free (SPF) facility with unrestricted access to water and food. The experimental animals were randomly divided into three groups (*n*=10/group). The control group received a normal rodent diet. The experimental autoimmune thyroiditis (EAT) group received 0.05% sodium iodide (0.64g/L NaI) water. The Allo-aca treatment group received NaI water and a subcutaneous injection of Allo-aca daily. 1mg/kg leptin receptor antagonist Allo-aca (MedChemExpress, Shanghai, China) was dissolved in 100µl saline and injected subcutaneously into the neck of the mice (19). After 8 weeks, the experimental animals were euthanized, and peripheral blood, thyroid was collected for subsequent experiments (**Figure 1**).

### Histological analysis of the thyroid gland

The spleen and thyroid gland were fixed in 4% paraformaldehyde solution, then embedded in paraffin, sectioned, and stained with hematoxylin and eosin. Experimental autoimmune thyroiditis was graded based on the percentage of lymphocytic infiltration in the thyroid gland by two researchers who observed the thyroid sections in a blinded fashion (20). Grade 0: normal thyroid; grade 1: lymphocytic infiltration in thyroid gland < 1%; grade 2: 1% ≤ lymphocytic infiltration < 10%; grade 3: 10% ≤ lymphocytic infiltration < 40%; grade 4: lymphocytic infiltration ≥40%.

### Enzyme-linked immunosorbent assay

Peripheral blood from mice was centrifuged at 3000 rpm for 20 minutes to separate serum. Serum levels of leptin, T3, T4, TSH, TPO-Ab, TG-Ab, IL-17 and IL-10 were measured on a microplate reader (Rayto RT-6100, Shenzhen, China) using the corresponding ELISA kits (FANKEL, Shanghai, China) following the manufacturer’s instructions. Thyroid tissue was completely homogenized, and the supernatant was separated by centrifugation at 3000 rpm for 20 minutes. Levels of STAT3, RORγt, Il-17, FOXP3, TGF-β, and IL -10 were detected in the thyroid using their respective ELISA kits.

### Multiplex fluorescence immunohistochemistry

Paraffin sections of thyroid tissue were dehydrated, antigen repaired, and blocked endogenous peroxidase activity and nonspecific antigens. Using the TSA tyramide signal amplification technique, samples were incubated with anti-FOXP3 antibody (Servicebio, Wuhan, China) and anti-IL-17A antibody (Proteintech, Wuhan, China) at 4 °C for 8 h, followed by the corresponding secondary antibody (Servicebio, Wuhan, China) were at room temperature for 50 min. The nuclei were labeled with DAPI (Servicebio, Wuhan, China) for 3 min. Recorded and photographed under a confocal laser microscope. The fluorescence intensities of FOXP3 and IL -17A were analyzed using ImageJ software.

### Statistical analysis

GraphPad Prism 8.0 was used for data analysis and charting. All data are presented as mean ± standard deviation (SD) or percent. Comparisons between two groups were performed with Student’s t-test; comparisons between multiple groups were performed with one-way analysis of variance (ANOVA). The 95% confidence level was considered significant. For all tests, statistical significance of p-values is shown as *ns*: not significant *, *P*< 0.05, **; *P* < 0.01; ***, *P* < 0.001; ****, *P* < 0.0001.

## Result

### Replication of EAT mouse model

**Figure 2:**
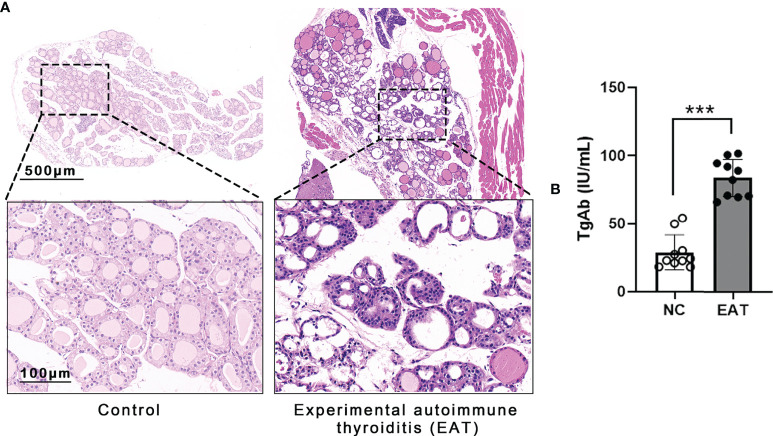
Experimental autoimmune thyroiditis (EAT) in mice. **(A)** The EAT mouse model was replicated by ingestion of 0.05% sodium iodine water. The thyroid follicular epithelial cells were necrotic, and a large number of lymphocytes infiltrated the thyroid gland of EAT group. **(B)** Compared with the NC group, the serum level of thyroid autoantibody TgAb was significantly increased in the EAT group. The above features indicate that the EAT model was successfully replicated. ***P < 0.001.

Experimental autoimmune thyroiditis (EAT) mouse model was induced by feeding the animal 0.05% NaI water for 8 weeks. Thyroid histology showed irregular morphology and destruction of thyroid follicles in EAT mice, and a massive number of lymphocytes infiltrated the thyroid gland (**Figure 2A**). The level of thyroglobulin antibodies (TgAb) in EAT mice elevated significantly compared with the control group (**Figure 2B**). These phenomena indicated that the EAT model is successfully replicated.

### Allo-aca attenuates thyroiditis in EAT mice

**Figure 3:**
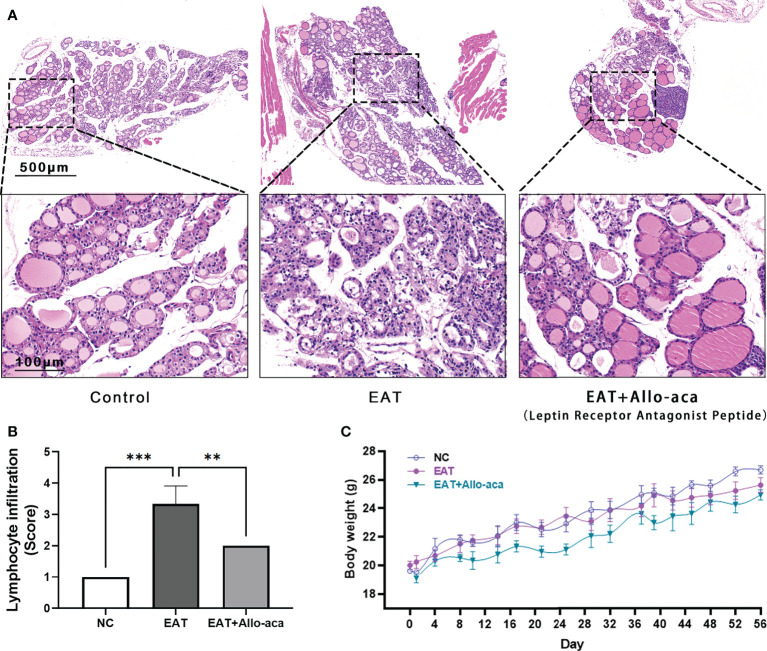
Allo-aca alleviates autoimmune thyroiditis caused by high iodine intake. **(A)** In normal mouse thyroid, the follicles were distributed evenly and there were no infiltrated inflammatory cells. High iodine intake caused autoimmune thyroiditis (EAT) in NOD mice, which was characterized by structural damage to thyroid follicles. Whereas treatment with Allo-aca attenuated the destruction of thyroid follicular cells. **(B)** Allo-aca significantly reduced the number of lymphocytes in the thyroid gland according to the lymphocyte infiltration score. **(C)** The body weight of mice was continuously monitored during the experimental period, and there was no statistical difference in body weight among the three groups. n = 4, data are expressed as mean ± standard deviation, **P < 0.01, ***P < 0.001.

The severity of thyroiditis in mice was assessed by the follicle morphology (**Figure 3A**) and lymphocyte infiltration rate (**Figure 3B**) in thyroid paraffin sections. Compared with the control group, the damage to the follicle and the lymphocyte infiltration rate were significantly increased in the EAT group, while these indicators were significantly decreased in the Allo-aca treatment group. Allo-aca showed no significant effect on the body weight of mice, and there was no statistical difference in body weight between the three groups (**Figure 3C**).

### Allo-aca down-regulated serum levels of T3, T4, TPOAb, and TgAb in EAT mice

**Figure 4:**
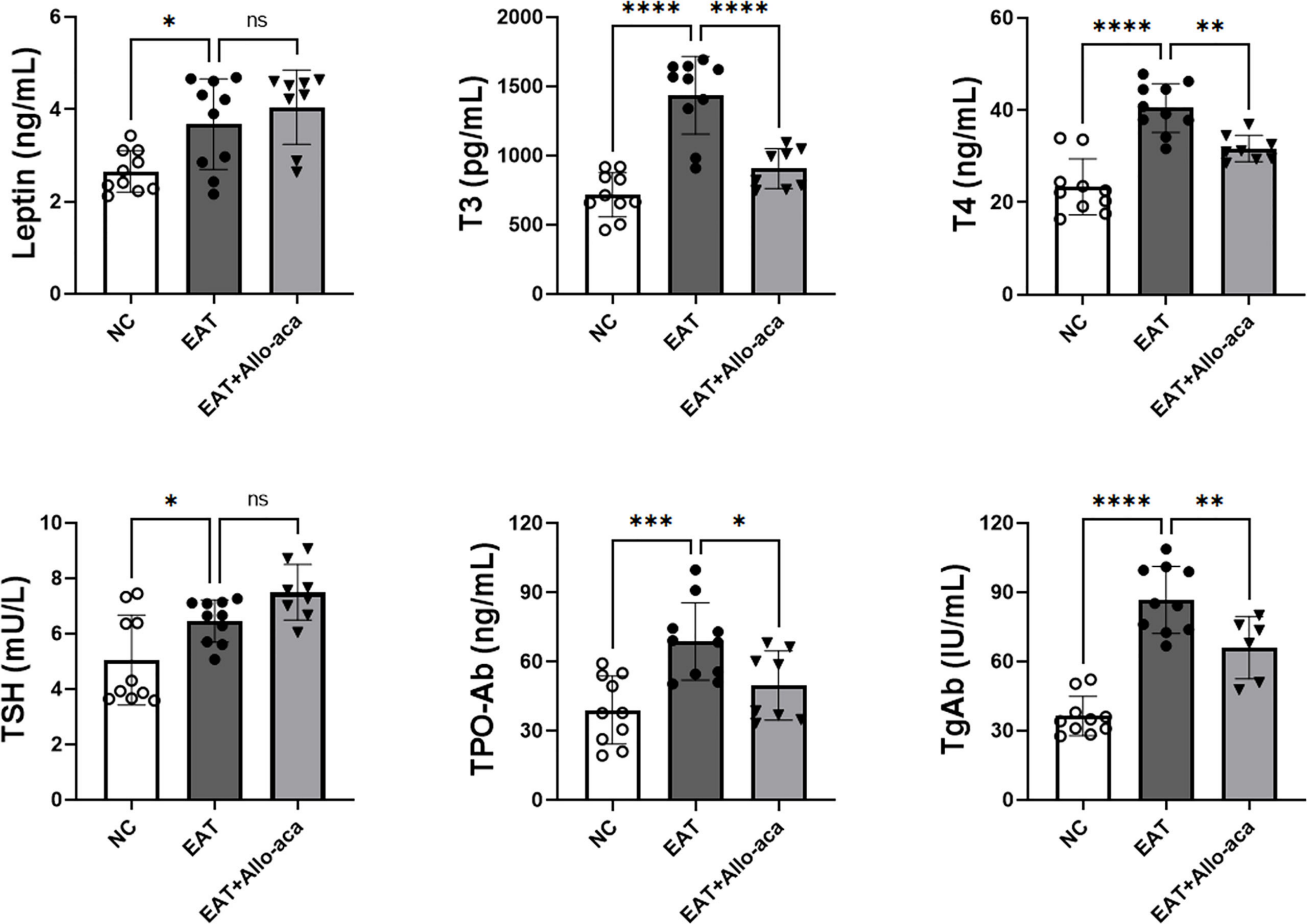
Serum leptin, thyroid hormone, and thyroid autoimmune antibody levels in experimental mice. Serum leptin, T3, T4, TSH, TPO-Ab, and TgAb expressions were all up-regulated in high iodine-induced EAT mice. Allo-aca reduced serum T3, T4, TPO-Ab, and TgAb levels in EAT mice. *n*=10, data are expressed as mean ± standard deviation, *ns*: not significant, *P < 0.05, **P < 0.01, ***P < 0.001, ****P < 0.0001.

The serum levels of leptin, T3, T4, TSH, TPOAb and TgAb were determined in the experimental animals by enzyme-linked immunosorbent assay (**Figure 4**). Compared with the control group, serum levels of leptin, T3, T4, TSH, TPOAb, and TgAb were all up-regulated in EAT mice. Allo-aca decreased the serum levels of T3, T4, TPO-Ab, and TgAb in EAT mice. A slight increase in serum TSH was observed in the Allo-aca treatment group, but it was not statistically significant (*P*=0.18).

### Allo-aca altered the proportion of Treg and Th17 cells in the thyroid gland of EAT mice

**Figure 5:**
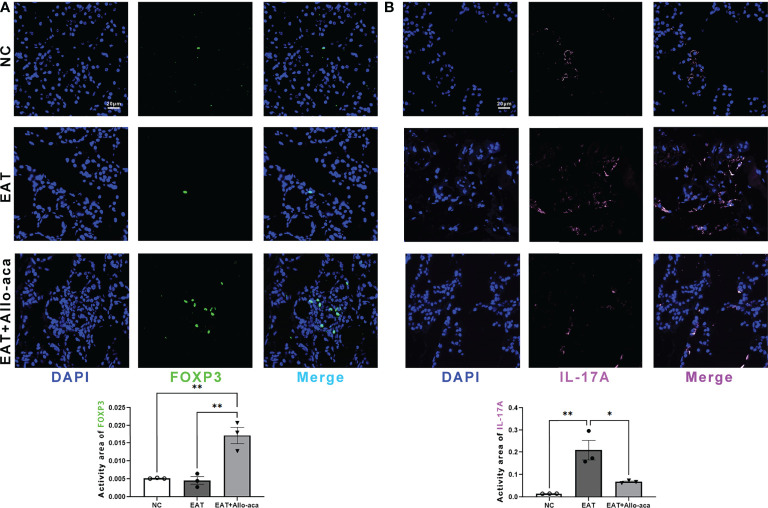
Allo-aca altered the expression of FOXP3 and IL -17A in the thyroid gland of EAT mice. **(A)** Allo-aca increased the level of FOXP3 in the thyroid gland of EAT mice. **(B)** The level of IL -17A was increased in the thyroid gland of EAT mice and was significantly attenuated by Allo-aca. FOX3P was labeled in green, IL -17A was labeled in pink, and nuclei were stained with DAPI. Scale bar: 20µm. *n*=3, data are expressed as mean ± standard deviation, *P < 0.05, **P < 0.01.

Multiplex fluorescence immunohistochemistry was used to detect Th17 cells and Treg cells in the thyroid gland of mice. Treg cells and Th17 cells are both derived from CD4-positive lymphocytes. Treg cells are characterized by FOXP3 positive and Th17 cells are characterized by IL -17A positive (21, 22). In this study, Allo-aca significantly increased the level of FOXP3 in the thyroid gland of EAT mice (**Figure 5A**). In contrast, the level of IL -17A significantly increased in the thyroid gland of EAT mice and was attenuated by Allo-aca (**Figure 5B**). The result showed that Allo-aca changed the proportion of Treg and Th17 cells, indicating the alleviation of thyroiditis.

### Allo-aca down-regulated the levels of STAT3, RORγt, Il-17, and up-regulated the level of FOXP3 in the thyroid gland of EAT mice

**Figure 6:**
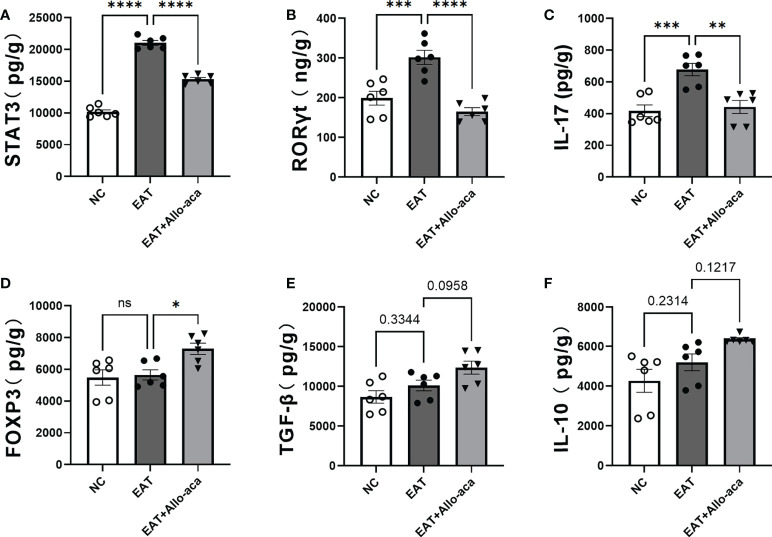
Effects of leptin receptor antagonists on inflammatory factors associated with hyper iodine-induced autoimmune thyroiditis in mice. **(A-C)**. The levels of STAT3, RORγt and IL-17 in thyroid of EAT mice increased and Allo-aca decreased their levels. **(D)**. Allo-aca increased the level of FOXP3 in thyroid of EAT mice. **(E-F)**. Allo-aca mildly increased the levels of TGF-β and IL-10 in thyroid of EAT mice, but the change was not statistically significant. *n*=6. Data is expressed as mean ± SEM. *ns*: not significant, *P < 0.05, **P < 0.01, ***P < 0.001, ****P < 0.0001.

ELISA was used to detect the levels of STAT3, RORγt, Il-17, FOXP3, TGF-β and IL-10 in mouse thyroid glands. In this study, the levels of STAT3, RORγt and Il-17 were up-regulated in EAT mice thyroid compared with controls. Allo-aca decreased the levels of STAT3, RORγt, and IL-17 in the thyroid gland of EAT mice (**Figures 6A–C**), and up-regulated the level of FOXP3 (**Figure 6D**). Allo-aca also slightly upregulated TGF-β and IL-10 levels in the thyroid glands of EAT mice, but not statistically significant (**Figures 6E, F**).

### Allo-aca upregulates serum IL-10 levels in EAT mice

**Figure 7:**
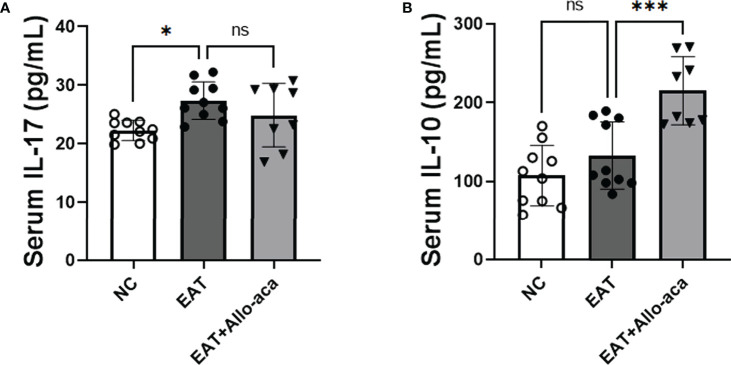
The levels of IL-17 and IL-10 in EAT mouse serum and the effect of Allo-aca on them. **(A)** Serum IL-17 levels in EAT mice were higher than in control mice, but Allo-aca had no significant effect on it. **(B)** Allo-aca significantly up-regulated serum IL-10 levels in EAT mice. *n*=10 in NC and EAT group, *n*=8 in Allo-aca group. Data is expressed as mean ± SEM. *ns*: not significant, *P < 0.05, ***P < 0.001.

In addition to the thyroid, we also detected the levels of inflammatory factors in the serum of experimental animals. IL-17 was up-regulated in the serum of EAT mice, and Allo-aca had no significant effect on it (**Figure 7A**). Allo-aca significantly up-regulated the level of serum IL-10 in EAT mice (**Figure 7B**).

## Discussion

Leptin has a role in promoting the development of inflammation in many autoimmune diseases such as gout (23), and osteoarthritis (24, 25). But the relationship of leptin to autoimmune thyroid disease has not been fully elucidated. Studies have found that serum leptin levels are slightly higher in patients with Hashimoto’s thyroiditis than in the normal group, but it is not statistically significant (26, 27). Postpartum thyroiditis patients are associated with significantly elevated leptin levels, and there may be an association between the two (28). Thyroid autoantibody levels were positively correlated with leptin levels in non-obese men, but this association was not found in women (29). In our study, serum leptin levels were increased and statistically significant in mice with experimental autoimmune thyroiditis.

Massive infiltration of T CD4+ lymphocytes is a key mechanism for thyroid cell destruction in autoimmune thyroid disease. And T CD4+ lymphocytes can differentiate into Th1, Th2, Th17 and Treg cells. The role of Th1 and Th2 cells in thyroiditis has been extensively studied. They mediate apoptosis/necrosis of thyroid follicular cells through cellular/humoral immune mechanisms (12, 13, 30, 31). In recent years, Th17/Treg cells have also been found to be important for thyroiditis, and several studies have shown that the imbalance of Th17/Treg cells is involved in the pathogenesis of autoimmune thyroid diseases (32–36). The increase in the number of Th17 cells in Hashimoto’s thyroiditis correlated positively with the degree of thyroiditis (37) and damage to the thyroid structure (38). Th17 cells stimulate macrophages, fibroblasts, and epithelial cells to produce cytokines by secreting IL -17, which leads to apoptosis of thyroid cells (39, 40). Blockade of IL -17 signaling significantly reduced lymphocyte infiltration in the thyroid gland of mice with experimental autoimmune thyroiditis (41). On the other hand, the number and function of Treg cells is reduced in autoimmune thyroiditis. Treg cells were supposed to reduce the inflammatory response in the thyroid gland by suppressing Th1, Th2, and Th17 cells (30, 42), but this effect was attenuated in AITD. In addition, Treg cells and Th17 cells inhibit each other’s differentiation (43, 44). Therefore, the ratio of Th17/Tregs is increased in AITD, which is critical for assessing the development of autoimmune thyroid disease.

**Figure 8:**
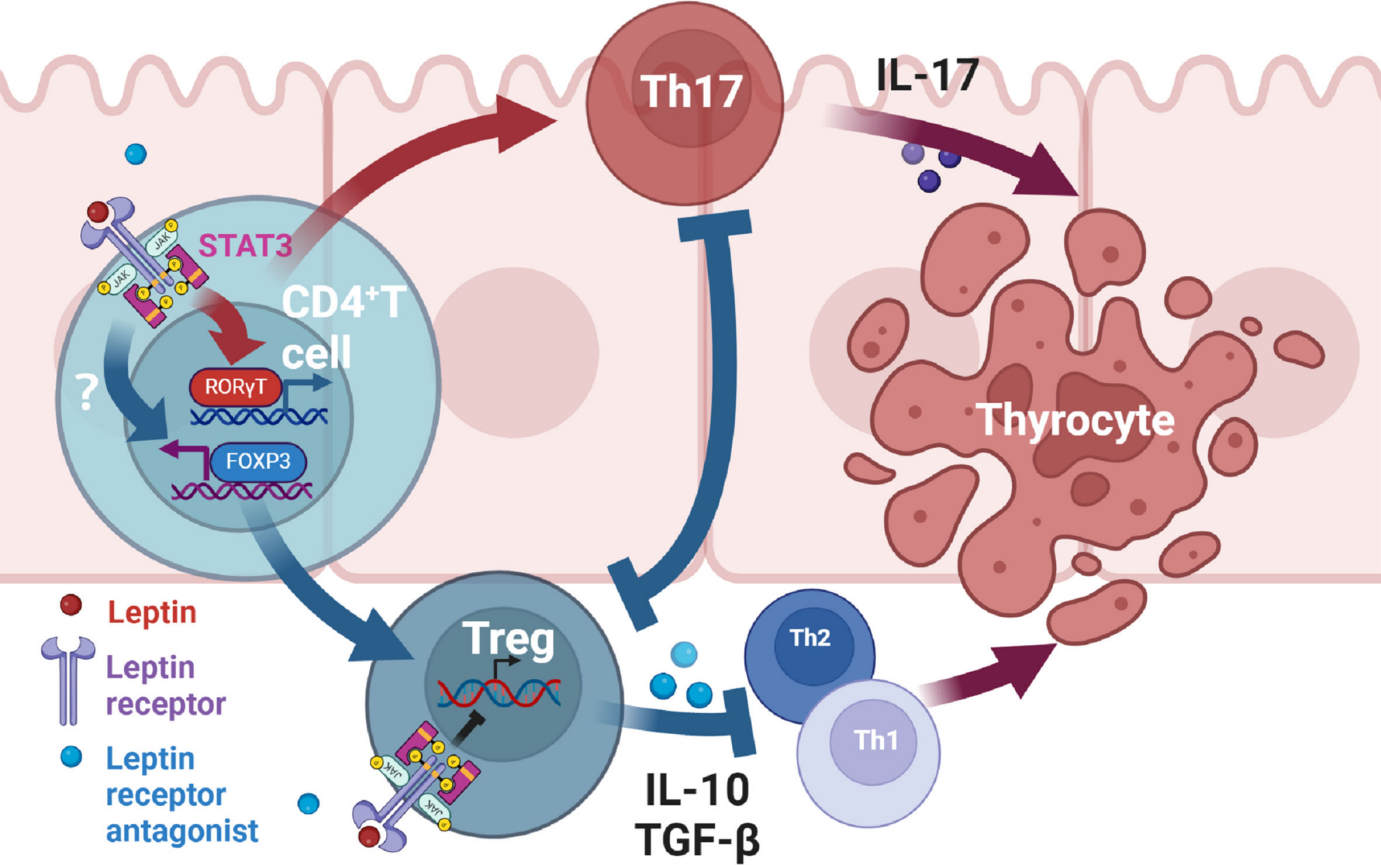
Mechanism of leptin receptor antagonists attenuating experimental autoimmune thyroiditis. Leptin binds to the leptin receptor (ObR) on the surface of T CD4+ lymphocytes, transmits signals to the nucleus through the JAK2/STAT3 pathway (47), promotes the transcription of RORγt, and then promotes the differentiation of T CD4+ lymphocytes into Th17 cells. Th17 cells mediate downstream immune responses through the production of IL-17, thereby leading to apoptosis/necrosis of thyroid cells. Treg can alleviate inflammatory response by inhibiting Th1, Th2 and Th17 cells. Treg and Th17 cells have antagonistic developmental programs and the differentiation of Treg inhibits Th17 differentiation (43, 44). On the other hand, Allo-aca can also bind to ObR of Treg cells to maintain Treg proliferation (46). Therefore, leptin receptor antagonists alleviate thyroid cell apoptosis/necrosis in experimental autoimmune thyroiditis by inhibiting Th17 cell differentiation and promoting Treg differentiation and proliferation.

There is evidence that leptin affects inflammation by altering the balance of Th17/Treg cells in autoimmune diseases (8, 43, 44). Leptin has been found to regulate the proliferation and differentiation of T lymphocytes. It increases Th1 and inhibits Th2 cytokine production (45). And leptin inhibits the proliferation of Treg cells, and this effect can be reinforced by the negative feedback generated by Treg cells autocreting leptin and overexpressing leptin receptors (46). Through this study, we suggest that antagonists of leptin receptors may alleviate thyroid damage in mice with experimental autoimmune thyroiditis by modulating Th17 and Treg cells. Serum leptin concentrations were higher in the EAT mice than in the normal mice. After administration of Allo-aca, an antagonist of the leptin receptor, thyroiditis in EAT mice was significantly alleviated, manifested as reduced thyroid follicle destruction, decreased lymphocyte infiltration, and decreased serum thyroid autoantibody levels. Thyroxine T3 and T4 tended to be normal, and TSH slightly increased compared with EAT model, but it was not statistically significant. Immunohistochemistry of the thyroid showed increased Treg cells and decreased Th17 cells in Allo-aca treated EAT mice. The levels of thyroid STAT3 (47), RORγt and IL-17 were decreased, the level of FOXP3 was increased, and the level of serum IL-10 was increased in Allo-aca treated EAT mice. In Hashimoto’s thyroiditis, T CD4+ lymphocytes, under the action of antigen presenting cells (APCs), activate RORγt through the JAK2/STAT3 pathway and differentiate into Th17 cells. This process requires the involvement of leptin, and Allo-aca blocks this process, thus inhibiting Th17 cell differentiation. On the other hand, blockade of the leptin pathway promoted the transcription of FOXP3 and induced the differentiation of T CD4+ lymphocytes into Treg cells. Since leptin has the effect of inhibiting the proliferation of Treg cells, blocking the leptin pathway also maintains the proliferation of Treg cells. Treg cells attenuated thyroid cell apoptosis/necrosis caused by autoimmune thyroiditis by inhibiting Th1, Th2 and Th17 (**Figure 8**).

## Conclusion

We found in the mouse model of autoimmune thyroiditis that antagonists of leptin receptors attenuated thyroid inflammation by promoting Treg cell differentiation and inhibiting Th17 cell differentiation. Therefore, intervening leptin signaling pathway may be a new approach to treat or improve Hashimoto’s thyroiditis.

## Data availability statement

The original contributions presented in the study are publicly available. This data can be found here: https://www.jianguoyun.com/p/DW3A6CIQ09n9Chi2jdwEIAA.

## Ethics statement

The animal study was reviewed and approved by The Animal Protection and Utilization Committee of Southwest Medical University.

## Author contributions

JJ and WW designed the research and wrote the manuscript. JJ and J-YX analyzed the data and reviewed the manuscript. B-TZ, Q-LJ, H-QZ, QX, and YZ performed the experiments. All authors reviewed the manuscript. All authors contributed to the article and approved the submitted version.

## Funding

This study was supported by the National Natural ScienceFoundation of China (82070288), The Science & Technology Department of Sichuan Province (22ZDYF3805), the Health Commission of Sichuan Province (21PJ100), The Office of Science and Technology and Intellectual Property of Luzhou (22YYJC0088), the Talent Development Project of The Affiliated Hospital of Southwest Medical University (20062), and the Affiliated Stomatological Hospital of Southwest Medical University (2022Y02).

## Conflict of interest

The authors declare that the research was conducted in the absence of any commercial or financial relationships that could be construed as a potential conflict of interest.

## Publisher’s note

All claims expressed in this article are solely those of the authors and do not necessarily represent those of their affiliated organizations, or those of the publisher, the editors and the reviewers. Any product that may be evaluated in this article, or claim that may be made by its manufacturer, is not guaranteed or endorsed by the publisher.
